# The Treatment of Lupus by Concentrated Light

**Published:** 1900-07-14

**Authors:** 


### Hospital Clinics and Medical Progress.

### THE TREATMENT OF LUPUS BY CONCENTRATED LIGHT.

One of the great drawbacks which has always stood in the way of treating microbial diseases by agents having a direct microbicidal action has been the unfortunate fact that most things which were strong enough to kill the microbes were also strong enough to kill or seriously injure the tissues in the midst of which they found their home. What was wanted was some agent which should exercise a selective affinity, something which should pick out the microbe, and, while killing it, should be harmless to the tissues around, and such an agent has been by no means easy to find. In fact, it has not been found. A near approach to it, however, seems to have been discovered in light, or, rather, in its violet and ultra-violet, or actinic rays, which have been shown to possess a marked bactericidal power. There would seem, then, to be everything in favour of the use of light as a means of killing the microbes which are the cause of lupus, if only the actinic rays can be made to penetrate sufficiently deeply, and the heat rays by which the tissues would be destroyed can be kept out. This Finsen has done. By passing the light through water, kept cool by a circulating arrangement, or through a bright blue solution of copper sulphate, also kept cool, most of the heat and red rays are absorbed, while by pressure upon the part to be operated on the tissues arerendered anæmic, so that the obstruction to the transmission of the actinic rays which would be caused by the presence of blood is much lessened. (See The Hospital, October 7th and December 9th.) Thus, it is possible to bring the influence of the concentrated actinic rays to bear upon microbes contained in the tissues even at a considerable depth from the surface. The results have seemed to fully bear out the anticipation, and there can be no doubt that in many of the cases treated by Dr. Finsen the affected tissue has appeared simply to revert to its natural healthy condition, leaving no scarring or deformity, which is what was wanted. With this we may be well content. There can be no doubt about the brilliancy of the results, and there is every reason to hope that now that the process is in full work at the London Hospital, we shall soon see amongst us living proof of the efficacy of the treatment. Whether it is wise, however, to jump to the conclusion that light kills the bacilli in the midst of a lupus nodule in the same direct manner as that in which it kills pathogenic organisms when thin plate cultures are exposed to its rays is another question. The production of sun-burn in those exposed to the light of the sun on glaciers, and of similar injury among men employed in various electro-metallurgical operations, is enough to show that light has a strong effect upon the tissues, stimulating them to new activities, or setting up in them inflammatory changes, or even destroying them; and in view of what we know about the manner in which living cells deal with pathogenic organisms, it seems quite as likely that the cure which follows on the exposure of lupus to light is the result of the inflammation or stimulation, or whatever we like to call the change which undoubtedly is thereby produced in the tissues, as by any direct microbicidal power which the light may possess. That this change is a great one there can be no question, resulting as it does in the production of redness and swelling, with serous discharge and sometimes the formation of bullæ.

According to the arrangements now in operation at the London Hospital for carrying out the treatment there are three sittings for patients every morning, and each sitting lasts an hour. A spot of the concentrated and cooled light about the size of a sixpence, is made to fall upon the skin at the point selected, and the skin at this point is kept in a comparatively bloodless condition by the nurse pressing the lower lens upon the skin with just enough force to keep the skin anæmic. It must be understood that the treatment has to be continued for a long time—many months, in fact, and that the applications have to be made daily, so that the whole business is somewhat tedious. The results, however, in the hands of Finsen, have in some cases been admirable.

